# Clinical trials registries are underused in the pregnancy and childbirth literature: a systematic review of the top 20 journals

**DOI:** 10.1186/s13104-016-2280-3

**Published:** 2016-10-21

**Authors:** Vadim V. Yerokhin, Branden K. Carr, Guy Sneed, Matt Vassar

**Affiliations:** 1 Oklahoma State University Center for Health Sciences, 1111 W. 17th St., Tulsa, OK 74107 USA; 2 St. John Clinic Administration, 1923 S. Utica, Davis Tower, ste 400, Tulsa, OK USA

**Keywords:** Publication bias, Systematic review, Clinical trials registries, Pregnancy and childbirth, Obstetrics

## Abstract

**Background:**

Systematic reviews and meta-analyses that do not include unpublished data in their analyses may be prone to publication bias, which in some cases has been shown to have deleterious consequences on determining the efficacy of interventions.

**Methods:**

We retrieved systematic reviews and meta-analyses published in the past 8 years (January 1, 2007–December 31, 2015) from the top 20 journals in the Pregnancy and Childbirth literature, as rated by Google Scholar’s h5-index. A meta-epidemiologic analysis was performed to determine the frequency with which authors searched clinical trials registries for unpublished data.

**Results:**

A PubMed search retrieved 372 citations, 297 of which were deemed to be either a systematic review or a meta-analysis and were included for analysis. Twelve (4 %) of these searched at least one WHO-approved clinical trials registry or clinicaltrials.gov.

**Conclusion:**

Systematic reviews and meta-analyses published in pregnancy and childbirth journals do not routinely report searches of clinical trials registries. Including these registries in systematic reviews may be a promising avenue to limit publication bias if registry searches locate unpublished trial data that could be used in the systematic review.

## Background

A systematic review is a form of research synthesis that brings together all available evidence using pre-determined methodologies to address a specific research question [1]. These reviews, when appropriate, may contain one or more meta-analyses whereby effect sizes from primary studies are combined statistically to produce a pooled effect estimate. For example, a recent systematic review of ten primary studies noted a twofold increase in perinatal depression in women with unexpected pregnancies [2]. Awareness of the association between depression and unplanned pregnancies can serve to lower clinical threshold for detection of depressive symptoms in new mothers, which can lead to timely and appropriate intervention. As such, systematic reviews and meta-analyses have the potential to ameliorate clinical practice and are of particular importance in a rapidly evolving specialty of pregnancy and childbirth.

While well-conducted systematic reviews are often considered the gold standard for determining care guidelines, they are susceptible to bias. One particular bias, known as publication bias, occurs when systematic reviews are comprised only of published studies with statistically significant outcomes. This bias likely misrepresents the true effectiveness of an intervention since only results showing significant differences are included. For instance, a study by Hart and colleagues [3] assessed systematic reviews and meta-analysis carried out on nine medications that were approved by the FDA in a single year: 2001. They found that when unpublished data were incorporated in these reviews, only 7 % of these meta-analyses predicted the drug in question to have the same efficacy. In other words, over 90 % of the systematic reviews carried out to make clinical decisions on interventions were incorrect as a result of publication bias. Publication bias is a known problem in maternal-foetal medicine research [4], and perinatology researchers should take precaution to limit this form of bias from systematic reviews. In a high pressure and high litigation field such as pregnancy and childbirth, where knowledge of the most current research advances is expected, the importance of highest quality evidence-based medicine cannot be overstated.

The primary means to limit publication bias is to use comprehensive and far-reaching search strategies to identify unpublished and non-significant data. While many data sources have been proposed, perhaps the most promising is to use clinical trials registries to locate unpublished trial data. These registries have been created across the globe, and the rate of clinical trial registrations is on the rise. For example, ClinicalTrials.gov received 206,176 registrations in 2015 alone [5]. In other words, this website receives 25 registrations per hour, 24 h per day, 365 days per year.

This astonishing volume of registrations is explained, in large part, by passage of section 801 of the Food and Drug Administration Amendment Act (FDAAA), which legally obligates registration of clinical trials meeting one or both of the following criteria prior to commencement of the trial:

“1. Trials of drugs and biologics: Controlled clinical investigations, other than phase 1 clinical investigations, of drugs or biological products subject to Food and Drug Administration (FDA) regulation.

2. Trials of devices: 1) Controlled trials with health outcomes of devices subject to FDA regulation, other than small feasibility studies, and 2) pediatric postmarket surveillance required by FDA”.

Despite the large number of registered clinical trials and strong recommendations from the Cochrane Collaboration to search trials registries for unpublished data, recent evidence suggests limited use of registries by systematic reviewers [6–9].

Here, we examine the prevalence of use of clinical trials registries searches by systematic reviewers in pregnancy and prenatal health journals. We also catalogue the specific registries searched and whether unpublished trial data were successfully found and/or incorporated into the systematic review findings. Finally, we examine the temporal trend of clinical trials registry searches over the past 8 years since passage of the FDA Amendments act mandated the registration of most clinical trials involving human patients prior to commencement.

## Methods

### Study design

This was a meta-epidemiologic systematic review, and thus registration with the international prospective register of systematic reviews (PROSPERO) did not apply. We identified the top 20 journals in the Pregnancy and Childbirth subspecialty of health and medical sciences using Google Scholar’s h5-index, which rates journals based on their “visibility and influence” [10]. Briefly, h5-index is an alternative to the traditional rating of scientific journals based on their “impact factor”, which takes into account the number of times an article is cited vs. the number of publication a journal produces [11]. The top 20 highest-rated journals in Pregnancy and Childbirth were searched for systematic reviews and meta-analyses published between January 1, 2007 and December 31, 2015. A search strategy was developed for high sensitivity and designed through collaboration with a National Institutes of Health medical librarian. The search was performed on December 29, 2015 and deployed as follows: ((((((((((((((((((((((((“Archives of disease in childhood. Fetal and neonatal edition”[Journal])) OR (“BMC pregnancy and childbirth”[Journal])) OR “Seminars in fetal & neonatal medicine”[Journal]) OR (“The journal of maternal-fetal & neonatal medicine: the official journal of the European Association of Perinatal Medicine, the Federation of Asia and Oceania Perinatal Societies, the International Society of Perinatal Obstetricians”[Journal])) OR “Journal of perinatology: official journal of the California Perinatal Association”[Journal]) OR (“Maternal and child health journal”[Journal])) OR (“Birth defects research. Part A, Clinical and molecular teratology”[Journal])) OR “Midwifery”[Journal]) OR “Seminars in perinatology”[Journal]) OR (“Paediatric and perinatal epidemiology”[Journal])) OR (“Fetal diagnosis and therapy”[Journal])) OR “Clinics in perinatology”[Journal]) OR “American journal of perinatology”[Journal]) OR “Journal of perinatal medicine”[Journal]) OR “Maternal & child nutrition”[Journal]) OR “Birth (Berkeley, Calif.)”[Journal]) OR “Birth defects research. Part C, Embryo today: reviews”[Journal]) OR “Journal of midwifery & women’s health”[Journal]) OR (“Journal of obstetric, gynecologic, and neonatal nursing: JOGNN/NAACOG”[Journal])) OR “Journal of human lactation: official journal of International Lactation Consultant Association”[Journal]) AND (((meta-analyses[Title/Abstract] OR meta-analysis[Title/Abstract] OR “meta analyses”[Title/Abstract] OR “meta analysis”[Title/Abstract] OR meta analyses[Title/Abstract] OR metaanalysis[Title/Abstract]) OR “systematic review”[Title/Abstract]) OR meta-analysis[Publication Type])) AND (“2007/01/01”[Date—Publication]: “2015/12/31”[Date—Publication])) AND “humans”[MeSH Terms]) NOT ((letter [pt] OR newspaper article [pt])). A more detailed search string, formatted in accordance with guidelines described in the Cochrane Handbook of Systematic reviews is publically available online (see “Availability of data and materials” section).

### Data extraction and training

Articles were retrieved using the search string above. Citations were imported and full text articles were retrieved using EndNote™ (Version X7). Each article not retrieved using this method was manually obtained by the authors through the home institution’s library subscriptions.

A training session was conducted during which a set of detailed steps for systematic data collection and analysis was explained and demonstrated to the team. The data of interest included the full names and abbreviations of each of the clinical trials registries. The methodology, which was based on searching each full text using the “Find” function, was verified against previously published data [9] and achieved 100 % accuracy, as compared to the original study.

### Screening and outcome measures

The authors (VY and BC) screened the title and abstract of all retrieved articles (N = 372) to determine if the citation met the criteria of a systematic review or meta-analysis. For the citations that likely did not meet the criteria, or if it was unclear whether or not the criteria were met, the full text of the study in question was carefully reviewed. Any disagreements were settled through a discussion between the authors. An article was classified as a systematic review if it met previously established criterion; specifically, articles were included if (1) the authors provided clear inclusion/exclusion criteria for the selected studies and (2) the authors attempted to perform a comprehensive search of the available literature on a pre-determined topic. A more detailed discussion on what constitutes a systematic reviews or meta-analysis can be found in previously published work [1, 12, 13].

Analogous to current publications on the topic [6, 8, 9], we chose to limit our search to the 16 World Health Organization (WHO)-approved registries given the stringent requirements for clinical trial registration maintained by these registries. We also included ClinicalTrials.gov, as it appears to be the most frequently searched clinical trials registry [6, 9]. The methods sections and any supplementary materials of each of the studies mentioning these clinical trials registries were carefully reviewed by Yerokhin and Carr to determine if the registry was searched, if usable data were found, and if the data were used for analysis in the publication. Finally, we chose to exclude Cochrane Central Register of Controlled Trials (CENTRAL) [14], as it is a collection of published clinical trials rather than a trials registry.

With help of medical librarians (JC and MF), we also checked whether or not applicable data were available on trials registry databases by searching for specific trials using the keywords provided by the systematic reviewers. This was accomplished by randomly choosing 26 systematic reviews and meta-analyses from our dataset of 297 studies (see below) included for analysis. Randomized selection was performed using the random number generator in Microsoft Excel. Two separate queries were deployed: one through clinicaltrials.gov and another through WHO-approved registries. The data were considered to be available, when a search query returned clinical trials with available data *prior* to publication of the review (e.g. if a review was published in 2014, only trials with data available on, or before, 2013 were considered applicable). This study’s protocol and manuscript creation was carried out in accordance to all applicable Preferred Reporting Items for Systematic Reviews and Meta-analyses (PRISMA) [15] guidelines.

## Results

**Fig. 1:**
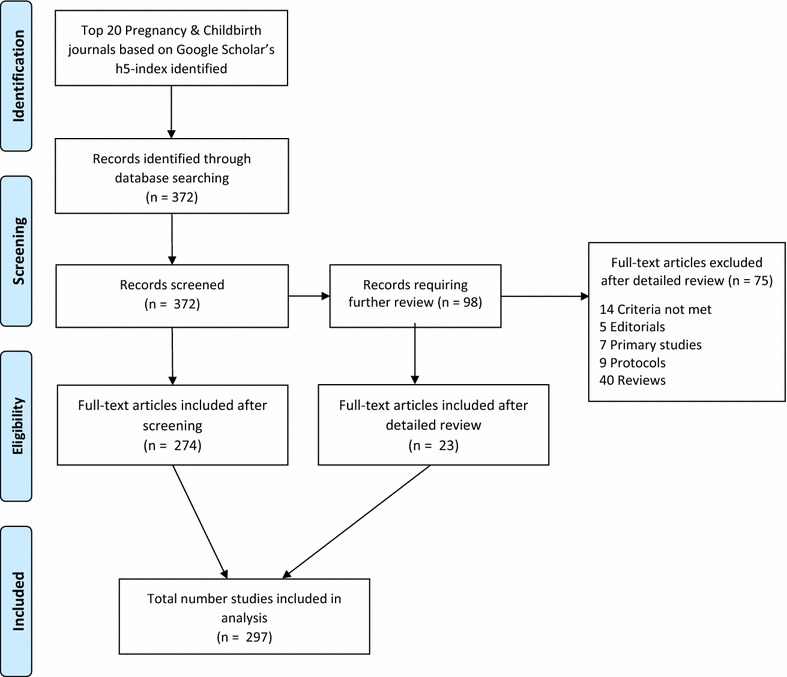
PRISMA flow diagram of selection process for analysis

Our PubMed search yielded a total of 372 articles published between January 1, 2007 and December 31, 2015. Of these, 297 publications were included for analysis (Fig. 1). A total of 75 studies were excluded from analysis because they either did not meet the criteria of a systematic review, or performed a pooled analysis of primary data from disease-specific databases. The main, coded dataset is publically available online (see “Availability of data and materials” section).

### Clinical trials registry search by journal

**Fig. 2:**
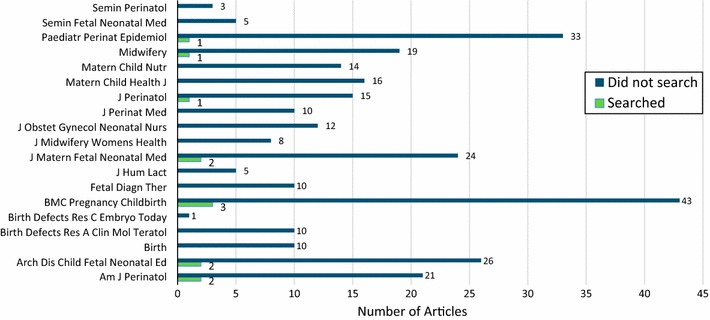
Frequency of clinical trials registry search by systematic reviews and meta-analyses published in the top 20 pregnancy and childbirth journals

The frequency of clinical trials registry searches was determined for each publication within the corresponding journal. Systematic reviews and meta-analyses published in *BMC Pregnancy and Childbirth*, *Paediatric Perinatal Epidemiology*, *Journal of Maternal Fetal and Neonatal Medicine*, *Archives of Disease in Childhood: Fetal and Neonatal Edition* and *American Journal of Perinatology* searched clinical trials registries most frequently. A total of 3 of 46 articles in *BMC Pregnancy and Childbirth*, 2 of 26 in *Journal of Maternal Fetal and Neonatal Medicine*, 2 of 23 articles in *American Journal of* Perinatology, and 1 of 34 in *Paediatric Perinatal Epidemiology* reported searches of clinical trials registries as part of the systematic review process. Systematic reviews retrieved from 12 of the 19 journals searched neither ClinicalTrials.gov, nor any of the 16 WHO-approved clinical trials registries (Fig. 2).

### Use of clinical trials registry data

We reviewed each study that searched any of the 17 clinical trials registries and determined if the study (1) found any applicable data and (2) used the data in their analysis. The findings are graphically represented in Table 1. The full text of each article was reviewed to determine if the authors indicated finding applicable data or using it. If this information was not explicitly provided in the article, we reviewed the author’s data tables (when available) and verified the included references to determine if any of the data used for analysis was retrieved from a clinical trials registry. A total of 12 studies [16–27] searched either ClinicalTrials.gov or the WHO-approved registries and one [22] of these reviews reported searches of both. Of these, two systematic reviews reported [16, 20] that applicable data were found, but neither of the systematic reviews used the data. Furthermore, 8 of the reviews did not clearly indicate whether or not data were found [17, 19, 21–26] and it was not possible to determine whether or not the data were used in 1 of the reviews [22]. In each of these cases, the corresponding cells are marked “yellow”. With the collected data in hand, two major questions remained unanswered for the majority of the articles: (1) if the authors searched clinical trials registry data, did they find any relevant data? and (2) if relevant data were found, did they include the data in their analysis? In an attempt to answer these questions, a contributing author (BC) contacted the corresponding authors of each of the publication included in Table 1 via email (see the “Availability of data and materials” section for the email template). Contacts attempts were made twice within a period of 14 days. We received a total of 4 (of 12) responses. One of the authors reported that although one applicable trial was found on a clinical trials registry, the trial was at the recruiting stage and did not have data available. The remaining three authors stated that data from clinical trials registries was not included because it was already published and included in the analysis dataset, dataset was missing or no unpublished findings met their inclusion criteria.

**Table 1:**
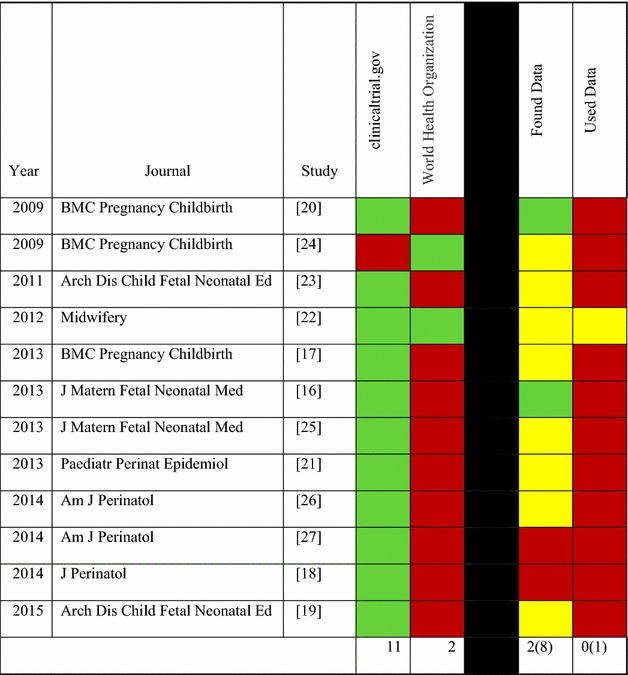
Grading chart of clinical trials registry utilization by systematic reviews and meta-analyses

*Green* yes, *Red* No, *Yellow* unclear

### Clinical trials registry search between 2007 and today

**Fig. 3:**
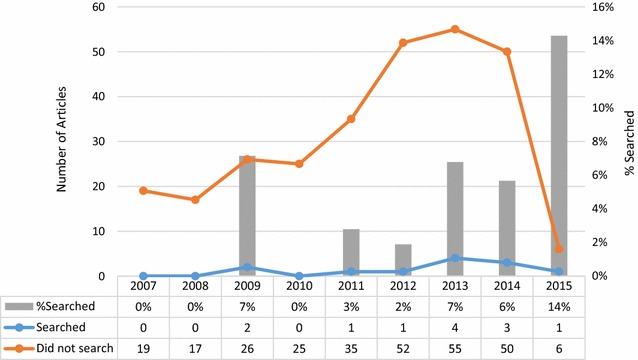
Temporal trend of clinical trials registry search by systematic reviews in the top 20 pregnancy and childbirth journals. The number (*left vertical axis*) of systematic reviews and meta-analyses searching (*blue line*) and not searching (*orange line*) clinical trials registries between 2007 and 2015. The *grey bars* represent the percentage (*right vertical axis*) of systematic reviews and meta-analyses that searched clinical trials registries for the given year

In 2006, the WHO established a set of 20 items that must be included for a clinical trial to register with the approved databases [28]. Among these items are requirements for submission of primary and key secondary trial outcomes. In an effort to make this data publically available, WHO also created the International Clinical Trials Registry Portal (ICTRP) [29], which can be searched by systematic reviewers for unpublished data. Similarly, the United States passed the Food and Drug Amendments Act of 2007 (FDAA) [30], setting a higher standard for clinical trial registration at ClinicalTrials.gov. To assess the effect these landmark decisions on use of clinical trials registries, we analysed the frequency with which these registries were searched by year. Interestingly, although there was an increase in systematic reviews and meta-analyses published since 2007, the proportion of these studies searching clinical trials registries did not appear to increase (Fig. 3). Because of indexing delays of published articles by PubMed, only seven systematic reviews were retrieved from the year of 2015, which is likely an underrepresentation of the total number published that year. Hence, it is difficult to draw any reliable conclusions about the number of systematic reviews and meta-analyses searching clinical trials registries that year.

### Potential data from clinical trials

As described above and discussed in further detail below, reliability of systematic reviews and meta-analyses in guiding clinical decision-making is dependent on retrieval of all applicable data. Studies published in other disciplines have previously shown that valuable unpublished data is available on clinical trials registries, providing support for the need to search clinical trials registries when designing and performing a systematic review [3, 8]. However, no study to date has attempted to determine the value of searching clinical trials registries in Pregnancy and Childbirth systematic reviews. Although it was not a primary outcome of this study, we searched the availability of data from clinical trials for 26 randomly selected systematic reviews. Clinicaltrials.gov (Table 2) was searched for trials with data applicable to each of the study’s respective topic. Our search indicated that a number of clinical trials on the database did, in fact, hold several studies with available data (yellow highlights), which could have been used by the systematic reviewers. In fact, a total of 190 clinical trials with available data were not included (or mentioned) during the systematic review. For instance, a search of clinicaltrials.gov for keywords in the systematic review “Vitamin A and Carotenoids During Pregnancy and Maternal, Neonatal and Infant Health Outcomes: A Systematic Review And Meta-Analysis” returned eight clinical trials (NCT00659061, NCT00715676, NCT01232205, NCT00363038, NCT00706004, NCT00493012, NCT00198822, NCT01198574), with a total of 61,228 patients enrolled. Each of these trials contained data, which should have been considered for, and could have potentially been used in, the systematic review.

**Table 2:** Availability of data from trials registered at clinicaltrials.gov

Journal	Article name	Search string	# applicable clinical trials retrieved	# of trials which could have potentially been included in the systematic review
J Obstet Gynecol Neonatal Nurs	A systematic review of the effectiveness of breastfeeding intervention delivery methods	(Breastfeeding OR lactation OR breast milk) AND (e-technologies OR intervention OR technology OR technologies OR web-based)	1655	0
Matern Child Nutr	Association between timing of introducing solid foods and obesity in infancy and childhood: a systematic review	(Infant OR newborn OR childhood OR infancy OR neonate OR neonatal) AND (feeding OR food OR breast milk OR breastfeeding OR solid food) AND (body mass index OR BMI OR obesity OR weight)	775	6
BMC Pregnancy Childbirth	Caesarean delivery and subsequent pregnancy interval: a systematic review and meta-analysis	(Caesarean section OR Caesarean delivery OR abdominal delivery OR C-section) AND (birth interval OR birth spacing OR pregnancy interval OR first birth interval OR inter-delivery interval OR pregnancy gap OR pregnancy spacing)	37	2
Paediatr Perinat Epidemiol	Effect of women’s nutrition before and during early pregnancy on maternal and infant outcomes: a systematic review	(Early pregnancy OR gestation OR pregnancy OR early gestation) AND (nutrition OR women’s nutrition OR diet) AND (outcome OR birth OR birth outcome OR birth outcomes)	597	9
Am J Perinatol	Endothelial nitric oxide synthase gene polymorphisms and risk of preeclampsia	(Preeclampsia OR pregnancy-induced hypertension OR gestational hypertension) AND (endothelial nitric oxide synthase OR endothelial NOS OR nitric oxide synthase)	3	0
Birth Defects Res A Clin Mol Teratol	Folic acid–containing supplement consumption during pregnancy and risk for oral clefts: a meta-analysis	(Cleft lip OR cleft palate OR oral cleft OR cleft) AND (folic acid OR Vitamin B9 OR B9 OR folate)	48	0
Birth Defects Res C Embryo Today	Genetic and nongenetic etiology of nonsyndromic anorectal malformations: a systematic review	(Anal atresia OR anorectal defect OR anorectal malformation OR birth defect) AND (genes OR genetic OR risk factors OR etiology)	2833	114
J Hum Lact	Immune markers in breast milk and fetal and maternal body fluids: a systematic review of perinatal concentrations	(Immune markers OR cytokine OR chemokine OR immunoglobulin) AND (breast milk OR amniotic fluid OR maternal serum OR cord serum OR saliva OR mucus)	424	2
J Midwifery Womens Health	Improving quality and safety in maternity care: the contribution of midwife-led care	(humanized care OR midwife OR physiologic birth OR home birth) AND (safety OR quality OR efficacy OR outcome)	1555	0
Arch Dis Child Fetal Neonatal Ed	Interleukin-6 (−174C) polymorphism and the risk of sepsis in very low birth weight infants: meta-analysis	(Infant, Newborn OR Infant, Premature OR Infant, Low Birth Weight) AND (cytokines OR interleukin-6 OR IL-6 OR genetics)	1183	0
BMC Pregnancy Childbirth	Key components of early intervention programs for preterm infants and their parents: a systematic review and meta-analysis	(Preterm infants OR preterm OR very low birthweight OR low birthweight OR premature) AND (intervention OR early intervention OR program OR guideline)	2661	3
Birth Defects Res A Clin Mol Teratol	Major, non-chromosomal, birth defects and maternal physical activity: a systematic review	(Physical activity OR exercise OR activity OR maternal exercise OR maternal physical activity) AND (birth defects OR defect)	2839	19
Matern Child Health J	Maternal mortality among migrants in western Europe: a meta-analysis	(Ethnic OR ethnic group OR migrant OR immigrant OR minority OR disparity OR ‘‘foreign nationality’’ OR ‘‘foreign nationals’’) AND (‘‘maternal mortality’’ OR ‘‘pregnancy-related mortality’’ OR ‘‘pregnancy-related death’’ OR ‘‘maternal deaths’’ OR maternal health)	66	1
Arch Dis Child Fetal Neonatal Ed	Music for medical indications in the neonatal period: a systematic review of randomised controlled trials	(Infant OR newborn OR neonate OR neonatal) AND (music OR music therapy)	25	0
Matern Child Health J	Nature or nurture: a systematic review of the effect of socio-economic status on the developmental and cognitive outcomes of children born preterm	((Preterm OR premature) AND (birth OR delivery OR infant)) OR (prematurity OR “low birth weight”) AND (social OR socioeconomic OR sociodemographic OR environment*) AND (intelligence OR “iQ” or cogniti* OR academic OR development)	2641	11
Arch Dis Child Fetal Neonatal Ed	Network meta-analysis of indomethacin versus ibuprofen versus placebo for PDA in preterm infants	(Infant OR newborn OR neonate OR neonatal) AND (ductus arteriosus OR Patent ductus OR PDA) AND (indomethacin OR ibuprofen OR cyclooxygenase inhibitors OR NSAID)	47	0
Arch Dis Child Fetal Neonatal Ed	Opioids for neonates receiving mechanical ventilation: a systematic review and meta-analysis	(Infant OR newborn OR neonate OR neonatal) AND (morphine OR diamorphine OR fentanyl OR alfentanil OR sufentanil OR pethidine OR meperidine OR codeine OR methadone OR narcotics) AND (mechanical ventilation OR ventilation OR respiration)	26	0
J Obstet Gynecol Neonatal Nurs	Parenting in the neonatal intensive care unit	(Parent OR parenting OR parents OR family OR family-centered care) AND (neonate OR neonatal intensive care unit OR NICU or intensive care OR ICU OR neonatal nursing)	644	0
Paediatr Perinat Epidemiol	Prenatal alcohol exposure and childhood balance: a systematic review	(Balance OR motor performance OR motor function OR coordination OR postural control OR posture) AND (child OR child development OR preschool child) AND (alcohol OR alcohol abuse) AND (maternal OR in utero OR pregnancy OR prenatal)	4	0
Midwifery	Psychosocial correlates of exclusive breastfeeding: a systematic review	(Breastfeeding OR breast feeding OR breast milk OR exclusive breastfeeding) AND (psychosocial OR duration OR predictors OR social OR support)	492	2
J Midwifery Womens Health	Results of microbial testing exploring the etiology of deep breast pain during lactation: a systematic review and meta-analysis of nonrandomized trials	(Breastfeeding OR lactation OR breast milk) AND (infection OR breast pain OR microbial OR mastitis OR nipple pain OR disease)	1262	4
Paediatr Perinat Epidemiol	Review of self-reported physical activity assessments for pregnancy: summary of the evidence for validity and reliability	(Physical activity OR exercise) AND (pregnancy OR gestation OR maternity) AND (questionnaire OR reliability OR validity OR review OR self-reported)	244	7
Paediatr Perinat Epidemiol	Systematic review and meta-analysis investigating breast feeding and childhood wheezing illness	(Breast-feeding OR milk, human OR infant formula OR bottle feeding) AND (asthma OR atopy OR atopic OR wheeze)	60	0
BMC Pregnancy Childbirth	Systematic review of clinical trials on dietary interventions to prevent excessive weight gain during pregnancy among normal weight, overweight and obese women	(Diet OR dietary intervention OR food intake OR eating habits) AND (gestational OR pregnancy) AND (weight OR weight gain)	314	2
Birth	Systematic review of the literature on postpartum care: effectiveness of interventions for smoking relapse prevention, cessation, and reduction in postpartum women	(Postpartum OR postpartum period OR neonate OR infant OR newborn) AND (smoking cessation OR maternal Smoking OR smoking)	127	0
Paediatr Perinat Epidemiol	Vitamin A and carotenoids during pregnancy and maternal, neonatal and infant health outcomes: a systematic review and meta-analysis	(Vitamin A OR carotenoids OR carotenoid) AND (pregnancy OR birth outcome OR infant OR growth OR mortality OR newborn OR neonate)	499	8

## Discussion

The goal of this study was to determine the frequency of clinical trials registry searches of systematic reviews and meta-analyses published in the highest-ranking Pregnancy and Childbirth journals. Our findings indicate that clinical trials registries continue to be widely underused in this specialty. Systematic reviewers are not using registries as a means to limit publication bias.

Given the broad scope of disease processes and the delicacy with which many clinical decisions in maternity and foetal care must be approached, systematic reviews and meta-analyses play a particularly important role in this specialty. By assimilating the most relevant primary research, systematic reviews and meta-analyses in the Pregnancy and Childbirth literature can be a useful tool for choosing an intervention that prioritizes “practices that are effective and least invasive, with limited or no known harms whenever possible” [31]. A fitting example is demonstrated in a systematic review performed by the Cochrane Collaboration Pregnancy and Childbirth Group, who summarized clinical trials assessing administration of corticosteroids to women at risk for pre-term birth [32]. Today, this routine intervention reduces infant mortality by 30–50 %.

Published in 1989, the book “Effective Care in Pregnancy and Childbirth” [33] was monumental in increasing availability and awareness of randomized trial evidence to pregnancy and maternal care physicians around the world. Since then, evidence-based medicine has become increasingly important for clinicians practicing in these specialties. The first large study to reveal the extent to which systematic reviews and meta-analyses influence clinical care in maternal and foetal medicine was performed by Wilson and colleagues [34] in 2002. The authors measured improvement in compliance with evidence-based medical guidelines across hospitals in United Kingdom in areas involving tissue closure, corticosteroid use for women at risk of pre-term birth, antibiotic prophylaxis for Caesarean section, and approaches to complicated vaginal birth. The authors found that since 1988, there was an average increase in compliance of 72, 82, 77 and 56 %, respectively for these specialties.

Today, the sheer volume of systematic reviews and meta-analyses published in the pregnancy and childbirth literature is remarkable. It is estimated that the majority (over 20 %) of all systematic reviews and meta-analyses present in medical literature are published in gynaecology, pregnancy and childbirth specialties [35]. Although these estimates are based on publications by the Cochrane Collaboration, others have also found that publications in obstetrics and gynaecology journals comprise a large portion of systematic reviews available [36]. As such, it should come as no surprise that systematic reviews and meta-analysis in Pregnancy and Childbirth have shaped essential clinical decisions, such as timing of corticosteroid administration for women at risk for preterm birth [32], methods of labour induction [37], approaches to intrapartum anaesthesia [38, 39], interventions for postpartum complications [40] and more [41–45]. With increased availability and use of systematic reviews and meta-analyses in making clinical decisions, it is essential that the quality of these works be maintained at the highest level. The movement to standardize and improve the quality of systematic reviews and meta-analyses in the obstetric literature has gained momentum in the past decade, as evidenced by the growing collection of publications on the topic [46–50]. Although increased standardization of systematic reviews and meta-analyses in the medical literature [51] has been improved, one aspect—methods to limit publication bias—continues to lag behind [6, 7, 9, 52–54].

As awareness of publication bias in systematic reviews increases [7, 55–58], we are only beginning to affirm the detrimental effects publication bias has on clinical practice [59–64]. In fact, a statement released in January 2016 by the International Committee of Medical Journal Editors (ICMJE) justly noted that “there is an ethical obligation to responsibly share data generated by interventional clinical trials because participants put themselves at risk.” [65]. An increased number of studies are finding that systematic reviews across various specialties, such as psychiatry [60, 62–64], oncology [59] and cardiorespiratory [61] may be providing erroneous conclusions as a result of publication bias.

This study has a number of limitations. For instance, we did not specifically examine the rates of trial registration by country or region. It is possible that registration rates differ between countries due to specific legislations. For example, passage of the FDA Amendments Act in the United States contributed to a sharp increase in trial registrations. In countries with no such legislation, trial registrations may be lower. The intent of our study was to examine rates of registry searching by systematic reviewers, and it is not known whether systematic reviewers from countries with such legislation in place would be more likely to search a clinical trials registry due to a greater awareness of their existence. This would be an interesting avenue for future research; however, such an investigation would be complicated by the number of international multi-center collaborations and the possibility for authors to register with a registry outside of their home country. Additionally, although it appears that for most of the studies clinical trials data was available, the actual inclusion criteria for the data from each trial would have been determined by the authors of the systematic review and hence, may not have been applicable to the study. Even so, none of the authors from the 26 randomly-selected reviews (see Table 2) reported searching or finding clinical trials data.

Finally, it is interesting to note that although we found that by searching clinical trials registries, over 50 % of systematic reviews could have obtained additional data, only a small fraction of the trials available on these registries reported the data, and could thus be used without the need to retrieve the data (Table 2). As such, it is possible that majority of authors may be discouraged from searching clinical trials registries, since the yield of available data is very low. If the case is such, we continue to strongly encourage the authors to search clinical trials registries for two reasons: (1) if time and monetary resources are an obstacle, it is still possible to set a filter to search only for trials, which contain data, hence, little effort is required to retrieve available data from trials registries and (2) it is possible to contact the research coordinator (whose contact information should always be listed on the study page) to ask for the missing data. Nonetheless, this may be a source of hesitation for authors and should thus be addressed in future research in order to perform a cost-benefit analysis for searching and attempting to retrieve the missing data from the registered clinical trials. We also encourage systematic reviewers to include more descriptive statements when reporting their data sources. Specifically, when a systematic reviewer does not indicate the source of retrieved data (as found in 7 of the 12 reviews listed in Table 1), it’s not possible to determine if the source of data was a clinical trials registry or a database of published works. One possible solution is to use the PRISMA guidelines for reporting of systematic reviews, which includes a template flow diagram [15].

## Conclusions

In conclusion, systematic reviewers in Pregnancy and Childbirth should search clinical trials registries to mitigate the implications of publication bias on the predicted efficacy of an intervention. Currently, the immediate consequences of publication bias on clinical decision-making in Pregnancy and Childbirth have yet to be fully understood. There is, however, increasing evidence that publication bias is present in the primary research [4], as well as systematic reviews and meta-analyses [14, 46–50] within the specialty. To our knowledge, this is the first study to undertake an assessment of this magnitude on the topic of publication bias in systematic reviews and meta-analyses in Pregnancy and Childbirth literature.

## Abbreviation

WHO: World Health Organization

## References

[1] Higgins J, Green S, Cochrane Collaboration (2011). Cochrane handbook for systematic reviews of interventions Chichester.

[2] Abajobir AA, Maravilla JC, Alati R, Najman JM (2016). A systematic review and meta-analysis of the association between unintended pregnancy and perinatal depression. J Affect Disord.

[3] Hart B, Lundh A, Bero L (2012). Effect of reporting bias on meta-analyses of drug trials: reanalysis of meta-analyses. BMJ.

[4] Blackwell SC, Thompson L, Refuerzo J (2009). Full publication of clinical trials presented at a national maternal-fetal medicine meeting: is there a publication bias?. Am J Perinatol.

[5] National Institutes of Health: Trends, charts, and maps. (2015). https://www.clinicaltrials.gov/ct2/resources/trends. Accessed 31 Dec 2015.

[6] Bibens ME, Chong AB, Vassar M (2016). Utilization of clinical trials registries in obstetrics and gynecology systematic reviews. Obstet Gynecol.

[7] Jones CW, Keil LG, Weaver MA, Platts-Mills TF (2014). Clinical trials registries are under-utilized in the conduct of systematic reviews: a cross-sectional analysis. Syst Rev.

[8] Keil LG, Platts-Mills TF, Jones CW (2015). Systematic reviews published in emergency medicine journals do not routinely search clinical trials registries: a cross-sectional analysis. Ann Emerg Med.

[9] Sinnett PM, Carr B, Cook G, Mucklerath H, Varney L, Weiher M, Yerokhin V, Vassar M (2015). Systematic reviewers in clinical neurology do not routinely search clinical trials registries. PLoS One.

[10] Google Scholar: Google Scholar Metrics. 2015. https://scholar.google.com/intl/en/scholar/metrics.html. Accessed 12 May 2016.

[11] Liu Z, Wan G. Comparing journal impact factor and H-type indices in virology journals. Library philosophy and practice (e-journal). Paper 891. 2012. http://digitalcommons.unl.edu/libphilprac/891.

[12] Geller NL, Proschan M (1996). Meta-analysis of clinical trials: a consumer’s guide. J Biopharm Stat.

[13] Crumley ET, Wiebe N, Cramer K, Klassen TP, Hartling L (2005). Which resources should be used to identify RCT/CCTs for systematic reviews: a systematic review. BMC Med Res Methodol.

[14] Ghanizadeh A, Sahraeizadeh A, Berk M (2014). A head-to-head comparison of aripiprazole and risperidone for safety and treating autistic disorders, a randomized double blind clinical trial. Child Psychiatry Hum Dev.

[15] Moher D, Liberati A, Tetzlaff J, Altman DG, The PRISMA Group (2009). Preferred reporting items for systematic reviews and meta-analyses: the PRISMA statement. PLoS Med.

[16] Al-Mandeel H, Alhindi MY, Sauve R (2013). Effects of intentional delivery on maternal and neonatal outcomes in pregnancies with preterm prelabour rupture of membranes between 28 and 34 weeks of gestation: a systematic review and meta-analysis. J Matern Fetal Neonatal Med.

[17] Bain ES, Middleton PF, Crowther CA (2013). Maternal adverse effects of different antenatal magnesium sulphate regimens for improving maternal and infant outcomes: a systematic review. BMC Pregnancy Childbirth.

[18] Bodnar LM, Pugh SJ, Abrams B, Himes KP, Hutcheon JA (2014). Gestational weight gain in twin pregnancies and maternal and child health: a systematic review. J Perinatol.

[19] Deshmukh M, Balasubramanian H, Rao S, Patole S (2015). Effect of gastric lavage on feeding in neonates born through meconium-stained liquor: a systematic review. Arch Dis Child Fetal Neonatal Ed.

[20] Ferrer P, Roberts I, Sydenham E, Blackhall K, Shakur H (2009). Anti-fibrinolytic agents in post partum haemorrhage: a systematic review. BMC Pregnancy Childbirth.

[21] Hackney DN, Olson-Chen C, Thornburg LL (2013). What do we know about the natural outcomes of preterm labour? A systematic review and meta-analysis of women without tocolysis in preterm labour. Paediatr Perinat Epidemiol.

[22] Hundley VA, Avan BI, Braunholtz D, Graham WJ (2012). Are birth kits a good idea? A systematic review of the evidence. Midwifery.

[23] Jones LJ, Craven PD, Attia J, Thakkinstian A, Wright I (2011). Network meta-analysis of indomethacin versus ibuprofen versus placebo for PDA in preterm infants. Arch Dis Child Fetal Neonatal Ed.

[24] Kidney E, Winter HR, Khan KS, Gulmezoglu AM, Meads CA, Deeks JJ, Macarthur C (2009). Systematic review of effect of community-level interventions to reduce maternal mortality. BMC Pregnancy Childbirth.

[25] Lindsay KL, Walsh CA, Brennan L, McAuliffe FM (2013). Probiotics in pregnancy and maternal outcomes: a systematic review. J Matern Fetal Neonatal Med.

[26] Ruifrok AE, van Poppel MN, van Wely M, Rogozinska E, Khan KS, de Groot CJ, Thangaratinam S, Mol BW (2014). Association between weight gain during pregnancy and pregnancy outcomes after dietary and lifestyle interventions: a meta-analysis. Am J Perinatol.

[27] Swaney P, Thorp J, Allen I (2014). Vitamin C supplementation in pregnancy—does it decrease rates of preterm birth? A systematic review. Am J Perinatol.

[28] World Health Organization: WHO Data Set. 2015. http://www.who.int/ictrp/network/trds/en/. Accessed 12 May 2016.

[29] World Health Organization: International Clinical Trials Registry Platform (ICTRP). 2016. http://www.who.int/ictrp/en/. Accessed 12 May 2016.

[30] National Institutes of Health: History, Policies and Law. 2015. https://www.clinicaltrials.gov/ct2/about-site/history. Accessed 3 June 2016.

[31] Sakala C, Corry MP (2008). Evidence-based maternity care: what it is and what it can achieve.

[32] Roberts D, Dalziel Stuart R. Antenatal corticosteroids for accelerating fetal lung maturation for women at risk of preterm birth. In: Cochrane database of systematic reviews. Hoboken: Wiley; 2006. 10.1002/14651858.CD004454.pub2 16856047

[33] Enkin M, Keirse M, Chalmers I (1989). Effective care in pregnancy and childbirth.

[34] Wilson B, Thornton JG, Hewison J, Lilford RJ, Watt I, Braunholtz D, Robinson M (2002). The Leeds University maternity audit project. Int J Qual Health Care.

[35] Dodd JM, Crowther CA (2006). Cochrane reviews in pregnancy: the role of perinatal randomized trials and systematic reviews in establishing evidence. Semin Fetal Neonatal Med.

[36] Kogan JR, Holmboe ES, Hauer KE (2009). Tools for direct observation and assessment of clinical skills of medical trainees: a systematic review. JAMA.

[37] Liu A, Lv J, Hu Y, Lang J, Ma L, Chen W (2014). Efficacy and safety of intravaginal misoprostol versus intracervical dinoprostone for labor induction at term: a systematic review and meta-analysis. J Obstet Gynaecol Res.

[38] Nicholson JM, Kellar LC, Henning GF, Waheed A, Colon-Gonzalez M, Ural S (2015). The association between the regular use of preventive labour induction and improved term birth outcomes: findings of a systematic review and meta-analysis. BJOG.

[39] Heesen M, Bohmer J, Klohr S, Hofmann T, Rossaint R, Straube S (2015). The effect of adding a background infusion to patient-controlled epidural labor analgesia on labor, maternal, and neonatal outcomes: a systematic review and meta-analysis. Anesth Analg.

[40] Hundley VA, Avan BI, Sullivan CJ, Graham WJ (2013). Should oral misoprostol be used to prevent postpartum haemorrhage in home-birth settings in low-resource countries? A systematic review of the evidence. BJOG.

[41] Furuta M, Sandall J, Bick D (2012). A systematic review of the relationship between severe maternal morbidity and post-traumatic stress disorder. BMC Pregnancy Childbirth.

[42] Vieira C, Portela A, Miller T, Coast E, Leone T, Marston C (2012). Increasing the use of skilled health personnel where traditional birth attendants were providers of childbirth care: a systematic review. PLoS One.

[43] Johnson MJ, Wootton SA, Leaf AA, Jackson AA (2012). Preterm birth and body composition at term equivalent age: a systematic review and meta-analysis. Pediatrics.

[44] Tura G, Fantahun M, Worku A (2013). The effect of health facility delivery on neonatal mortality: systematic review and meta-analysis. BMC Pregnancy Childbirth.

[45] Malin GL, Morris RK, Riley R, Teune MJ, Khan KS (2014). When is birthweight at term abnormally low? A systematic review and meta-analysis of the association and predictive ability of current birthweight standards for neonatal outcomes. BJOG.

[46] Windsor B, Popovich I, Jordan V, Showell M, Shea B, Farquhar C (2012). Methodological quality of systematic reviews in subfertility: a comparison of cochrane and non-cochrane systematic reviews in assisted reproductive technologies. Hum Reprod.

[47] Al Faleh K, Al-Omran M (2009). Reporting and methodologic quality of Cochrane Neonatal Review Group systematic reviews. BMC Pediatr.

[48] Smith V, Devane D, Begley CM, Clarke M, Higgins S (2007). A systematic review and quality assessment of systematic reviews of fetal fibronectin and transvaginal length for predicting preterm birth. Eur J Obstet Gynecol Reprod Biol.

[49] Bonfill X, Roque M, Aller MB, Osorio D, Foradada C, Vives A, Rigau D (2013). Development of quality of care indicators from systematic reviews: the case of hospital delivery. Implement Sci.

[50] Morris RK, Selman TJ, Zamora J, Khan KS (2011). Methodological quality of test accuracy studies included in systematic reviews in obstetrics and gynaecology: sources of bias. BMC Womens Health.

[51] Tunis AS, McInnes MD, Hanna R, Esmail K (2013). Association of study quality with completeness of reporting: have completeness of reporting and quality of systematic reviews and meta-analyses in major radiology journals changed since publication of the PRISMA statement?. Radiology.

[52] Kong Y, Wei X, Duan L, Wang W, Zhong Z, Ming Z, Zeng R (2015). Rating the quality of evidence: the GRADE system in systematic reviews/meta-analyses of AKI. Ren Fail.

[53] Tashani OA, El-Tumi H, Aneiba K (2015). Quality of systematic reviews: an example of studies comparing artificial disc replacement with fusion in the cervical spine. Libyan J Med.

[54] Kringos DS, Sunol R, Wagner C, Mannion R, Michel P, Klazinga NS, Groene O, Consortium DU (2015). The influence of context on the effectiveness of hospital quality improvement strategies: a review of systematic reviews. BMC Health Serv Res.

[55] Thaler K, Kien C, Nussbaumer B, Van Noord MG, Griebler U, Klerings I, Gartlehner G, Consortium UP (2015). Inadequate use and regulation of interventions against publication bias decreases their effectiveness: a systematic review. J Clin Epidemiol.

[56] Jones CW, Handler L, Crowell KE, Keil LG, Weaver MA, Platts-Mills TF (2013). Non-publication of large randomized clinical trials: cross sectional analysis. BMJ.

[57] Jones CW, Keil LG, Holland WC, Caughey MC, Platts-Mills TF (2015). Comparison of registered and published outcomes in randomized controlled trials: a systematic review. BMC Med.

[58] Kien C, Nussbaumer B, Thaler KJ, Griebler U, Van Noord MG, Wagner P, Gartlehner G, Consortium UP (2014). Barriers to and facilitators of interventions to counter publication bias: thematic analysis of scholarly articles and stakeholder interviews. BMC Health Serv Res.

[59] Burdett S, Stewart LA, Tierney JF (2003). Publication bias and meta-analyses: a practical example. Int J Technol Assess Health Care.

[60] Howland RH (2011). Publication bias and outcome reporting bias agomelatine as a case example. J Psychosoc Nurs Men.

[61] LeVois ME, Layard MW (1995). Publication bias in the environmental tobacco smoke/coronary heart disease epidemiologic literature. Regul Toxicol Pharmacol.

[62] Turner EH, Knoepflmacher D, Shapley L (2012). Publication bias in antipsychotic trials: an analysis of efficacy comparing the published literature to the US Food and Drug Administration database. PLoS Med.

[63] Turner EH, Matthews AM, Linardatos E, Tell RA, Rosenthal R (2008). Selective publication of antidepressant trials and its influence on apparent efficacy. N Engl J Med.

[64] Whittington CJ, Kendall T, Fonagy P, Cottrell D, Cotgrove A, Boddington E (2004). Selective serotonin reuptake inhibitors in childhood depression: systematic review of published versus unpublished data. Lancet.

[65] Taichman DB, Backus J, Baethge C, Bauchner H, de Leeuw PW, Drazen JM, Fletcher J, Frizelle FA, Groves T, Haileamlak A (2016). Sharing clinical trial data—a proposal from the international committee of medical journal editors. N Engl J Med.

